# Positive cofactor 4 (PC4) contributes to the regulation of replication-dependent canonical histone gene expression

**DOI:** 10.1186/s12867-018-0110-y

**Published:** 2018-07-27

**Authors:** Aleksandra Brzek, Marlena Cichocka, Jakub Dolata, Wojciech Juzwa, Daniel Schümperli, Katarzyna Dorota Raczynska

**Affiliations:** 1 Department of Gene Expression, Institute of Molecular Biology and Biotechnology, Adam Mickiewicz University in Poznan, Wieniawskiego 1, 61-712 Poznan, Poland; 2 Department of Biotechnology and Food Microbiology, Poznan University of Life Sciences, Wojska Polskiego 28, 60-637 Poznan, Poland; 3 Institute of Cell Biology, University of Bern, Baltzerstrasse 4, 3012 Bern, Switzerland

**Keywords:** PC4 transcriptional coactivator, Replication-dependent histones, Cell cycle, RNAP2 recruitment, 3′ end processing efficiency, U7 snRNP

## Abstract

**Background:**

Core canonical histones are required in the S phase of the cell cycle to pack newly synthetized DNA, therefore the expression of their genes is highly activated during DNA replication. In mammalian cells, this increment is achieved by both enhanced transcription and 3′ end processing. In this paper, we described positive cofactor 4 (PC4) as a protein that contributes to the regulation of replication-dependent histone gene expression.

**Results:**

We showed that PC4 influences RNA polymerase II recruitment to histone gene loci in a cell cycle-dependent manner. The most important effect was observed in S phase where PC4 knockdown leads to the elevated level of RNA polymerase II on histone genes, which corresponds to the increased total level of those gene transcripts. The opposite effect was caused by PC4 overexpression. Moreover, we found that PC4 has a negative effect on the unique 3′ end processing of histone pre-mRNAs that can be based on the interaction of PC4 with U7 snRNP and CstF64. Interestingly, this effect does not depend on the cell cycle.

**Conclusions:**

We conclude that PC4 might repress RNA polymerase II recruitment and transcription of replication-dependent histone genes in order to maintain the very delicate balance between histone gene expression and DNA synthesis. It guards the cell from excess of histones in S phase. Moreover, PC4 might promote the interaction of cleavage and polyadenylation complex with histone pre-mRNAs, that might impede with the recruitment of histone cleavage complex. This in turn decreases the 3′ end processing efficiency of histone gene transcripts.

**Electronic supplementary material:**

The online version of this article (10.1186/s12867-018-0110-y) contains supplementary material, which is available to authorized users.

## Background

Histones organize chromatin structure by forming a skeleton for DNA and therefore are crucial for cell viability. There are four core histones, H2A, H2B, H3 and H4, which together with 147 base pairs of DNA create an octameric core called nucleosome. The linker histone H1 associates with the DNA between two neighboring nucleosomes. A complete new set of histones is necessary in every cell division to pack newly synthesized DNA. However, after this process, the availability of histones must be reduced, because their excess could be harmful to the cell. Thus, histone protein synthesis is strictly coupled to DNA synthesis in the S phase of the cell cycle. These two processes are finely balanced as any disturbance may result in cell cycle arrest, increased DNA damage sensitivity and chromosome instability which, in consequence, may lead to developmental failure [1–3]. Genes that encode for histone variants that are expressed in terminally differentiated cells and not in S phase are not influenced by this regulation. These replication-independent histones (RIH) are incorporated into core particles to compensate for the histones that have been displaced as a result of active transcription of certain genes or DNA damage [4].

During the G1/S phase transition, the expression of replication-dependent histone (RDH) genes is highly activated, and histone mRNA levels increase ~ 35-fold due to a combination of activated transcription, efficient 3′ end processing and increased transcript stability. Then, at the end of S phase, RDH gene expression rapidly drops and stays at basal level during other phases of the cell cycle [2, 5]. Such a tight regulation of RDH gene expression requires many factors (for a review, see ref. [6]). Among them, the general transcription cofactor nuclear protein, ataxia-telangiectasia locus (NPAT) activates transcription of RDH genes at the onset of S phase by ~ 5-fold through interaction with other histone-specific transcription factors [2, 7, 8]. On the other hand, the efficiency of histone mRNA 3′ end processing increases ~ 8-fold during the G1*/*S phase transition [2]. Interestingly, replication-dependent histone mRNAs in metazoans are unique as they are the only known protein-coding transcripts that are not polyadenylated. These non-polyadenylated transcripts contain elements which are recognized by specialized factors that mediate the 3′ end processing by single cleavage via the endonuclease, CPSF73 [9–11]. A crucial role in 3′ end processing of histone mRNAs is played by the U7 snRNP (U7 small nuclear ribonucleoprotein). The U7 snRNP complex interacts with histone pre-mRNAs by RNA:RNA base-pairing of U7 snRNA and the histone downstream element (HDE), which is located few nucleotides downstream of the cleavage site [9, 12–14]. Then, Lsm11—one of two U7 snRNP-specific proteins [14, 15]—directly interacts with FLASH (FLICE-associated huge protein) to form a binding platform for recruitment of a heat-labile processing factor (HLF), that contains symplekin, CstF64 and other components of the cleavage and polyadenylation machinery (CPA), including the endonuclease CPSF73 [11, 16–18].

CstF64 is the only cell-cycle regulated factor shared between the histone pre-mRNA cleavage complex (HCC) and the CPA complex. During the cell cycle its expression profile parallels the upregulation of histone RNA processing [19]. It is suggested that by changing the interacting partners, CSTF64 may modulate the specificity of the resulting complex for a particular processing reaction. In G1 phase the amount of CstF64 is limited and it might be efficiently recruited by CstF77 and included in the CPA complex. Elevated concentration of CstF64 towards G1/S phase transition might favor an interaction with symplekin, resulting in tethering the HCC and its catalytic endonuclease CPSF73 to the factors already bound to histone pre-mRNA, i.e. FLASH and the U7 snRNP [19].

In 2001, Calvo and Manley showed that CstF64 interacts with Positive Coactivator 4 (PC4; also known as p15 or SUB1) [20]. PC4 is a nuclear protein, mostly known as a co-activator that markedly enhances transcription of class II genes [21, 22]. This coactivator’s function is associated with dsDNA binding affinity and both abilities are negatively regulated by PC4 phosphorylation [21]. In turn, the phosphorylated form of PC4 preferentially binds to ssDNA structures and this is associated with PC4 transcriptional repression activity [23–26]. Interestingly, in proliferating mammalian cells, the great majority (around 95%) of the total cellular PC4 is phosphorylated [22], and this form of PC4 interacts with CstF64 [20].

In this paper, we describe PC4 as a factor that might contribute to the regulation of replication-dependent histone gene expression. In chromatin immunoprecipitation (ChIP) experiments, we showed that PC4 might influence RNA polymerase II (RNAP2) interaction with RDH genes in a cell cycle-dependent manner. The most important effect was observed in S phase-synchronized cells, where PC4 knockdown leads to the elevated levels of RNAP2 on RDH histone genes, which corresponds to the increased total level of RDH gene transcripts. PC4 overexpression causes the opposite effect. These results suggest that PC4 might play a role as a negative factor involved in maintaining the very delicate balance between RDH gene expression and DNA synthesis during the cell cycle. Moreover, we found that PC4 can also be involved in the 3′ end processing of RDH gene pre-mRNAs. We noticed elevated levels of extended, incorrectly processed RDH gene transcripts in cells overexpressing PC4 and, conversely, elevated levels of correctly cleaved transcripts in cells with PC4 knockdown. This effect did not depend on the cell cycle and suggests that PC4 might promote the interaction of cleavage factors with RDH pre-mRNAs. The activity of PC4 on the transcription of RDH genes and the 3′ end processing of their transcripts may be mediated by its interaction with CstF64 and U7 snRNP.

## Results

### PC4 interacts with the U7 snRNP complex

Affinity chromatography based on MS2-tagged U7 snRNA followed by mass spectrometry analysis was performed in order to identify new proteins interacting with U7 snRNP. The procedure, described in details in our previous paper [27], led to the identification of PC4 as a new factor that could be involved in the regulation of canonical histone gene expression. PC4 protein was identified in a total of 8 different U7-enriched fractions obtained by various methods (see Additional file 1: Table S1). PC4 was also identified once in a negative control, that probably resulted from an unspecific interaction with the affinity column (MS2-MBP bound to amylose beads).

### PC4 affects RNAP2 occupancy on RDH genes

To elucidate the role of PC4 in replication-dependent histone gene expression, we prepared a HeLa cell line with stable overexpression of N-terminally FLAG-tagged PC4 (PC4 OE) (see Additional file 2: Figure S1A, B) and a HeLa cell line with PC4 knockdown (PC4 KD) using doxycycline-inducible production of siRNA that targets PC4 mRNA. In the latter case, cells were treated by doxycycline for 3 days which led to a reduction of PC4 mRNA and protein to approximately 12 and 26% of normal levels, respectively (see Additional file 2: Figure S1C, D). HeLa scramble cells which express a siRNA that does not hybridize to any known human mRNA were used as a negative control in RT-qPCR analysis (see Additional file 2: Figure S1C).

PC4 is a known transcriptional co-activator of RNAP2 [21, 22]. Therefore, we wondered whether it might influence RNAP2 occupancy on replication-dependent histone genes. To answer this question, we performed ChIP-seq (ChIP followed by high throughput sequencing) with anti-RNAP2 antibody using PC4 OE cells and EBFP OE cells (as a negative control). Moreover, for this experiment cells were synchronized to G1 and S phase in order to describe the influence of PC4 on RDH gene transcription during the cell cycle. The synchronization efficiency is summarized in Additional file 3: Figure S2A. As control genes, we used replication-independent histones genes, whose expression does not change during the cell cycle.

**Fig. 1:**
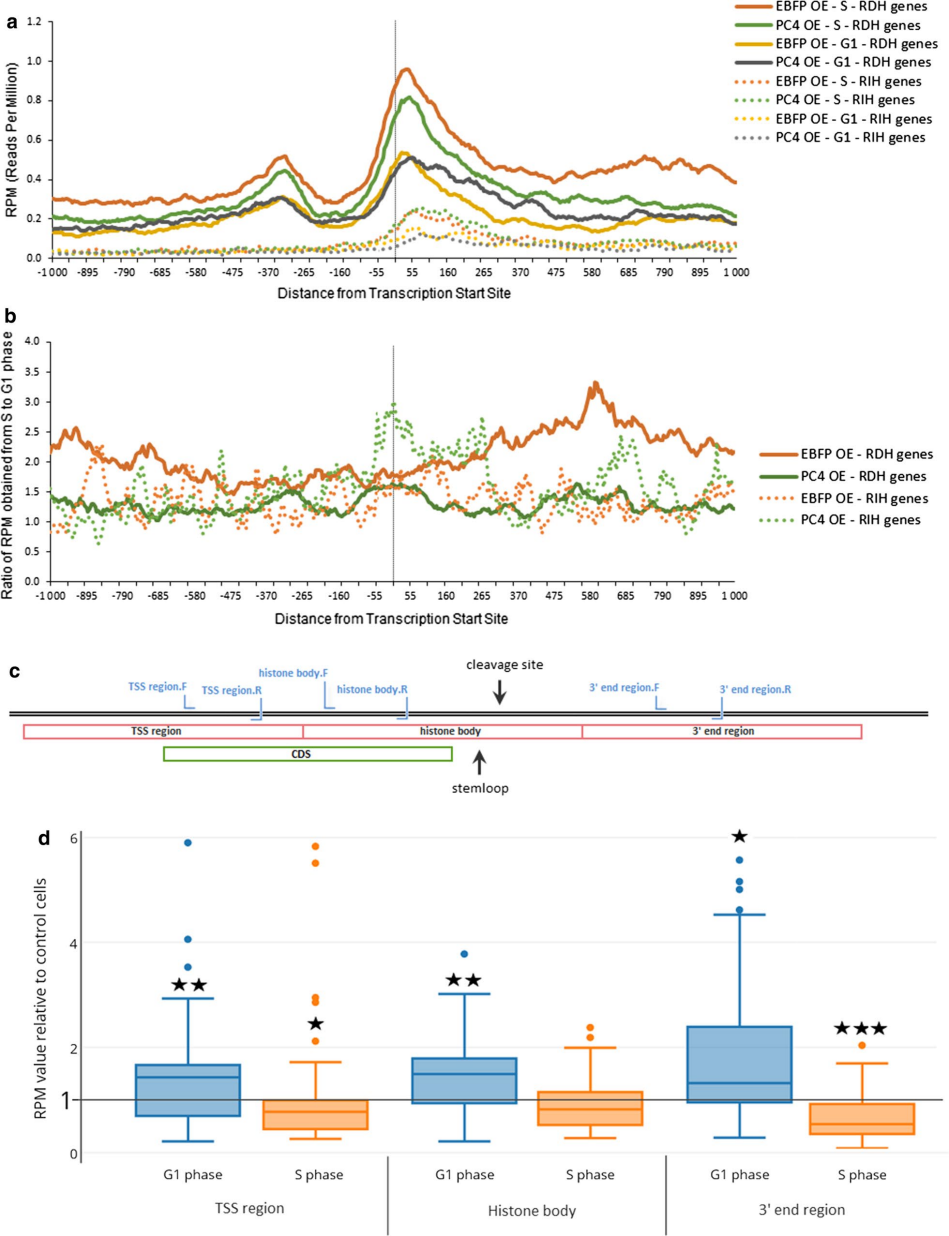
ChIP-seq analysis of PC4 overexpressing cells synchronized to G1 and S phase revealed changes in RNAP2 occupancy on RDH genes. **a** Profile of RNAP2 occupancy on all RHD and RIH loci in the range of 1000 base pairs upstream and 1000 base pairs downstream of the transcription start site (TSS, marked by vertical line). **b** Panel representing the ratio between reads per million (RPM) obtained from S phase to RPM obtained from G1 phase called “transcription activation in S phase” factor; TSS marked by vertical line. **c** A diagram of a RDH gene (an example on HIST1H3D) showing the location of primers used and regions analyzed in ChIP experiments. **d** Box plot of RNAP2 occupancy on 57 (out of 68) RDH genes in 400 nucleotides-long ranges: “TSS region”, “histone body” and “3′ end” in G1 phase- and S phase-synchronized cells with PC4 overexpression. Error bars indicate standard deviations (SD) of three biological replicates. P-values were calculated using Student’s T-test and statistical significance is represented as follows: *P ≤ 0,1; **P ≤ 0,05; ***P ≤ 0,003

In these ChIP-seq experiments, the RNAP2 occupancy was analyzed in the range of 1000 nucleotides upstream and downstream of the Transcription Start Site (TSS) of RDH genes. This region encompasses the 5′UTR with promoter sequence, coding region (body) and 3′UTR of the RDH genes. In the case of PC4 overexpression, we observed a diminished occupancy of RNAP2 on RDH genes in S phase compared to control cells overexpressing EBFP (Fig. 1a), whereas in G1 phase RNAP2 occupancy on these genes was similar or slightly enhanced. In the same experiments, RNAP2 distribution on replication-independent histone genes was not changed during the cell cycle. For a better estimation, we analyzed then the ratio between reads per million (RPM) obtained from cells synchronized to S phase to RMP obtained from cells synchronized to G1 phase, and we called it “transcription activation in S phase” factor. In control cells, this mean factor is 2.1 (median 2.0), according to the higher expression of canonical histone genes in S phase, however in PC4 overexpressing cells this value decreases to 1.3 (median 1.3) along the length of RDH genes (Fig. 1b), suggesting that PC4 might influence, directly or indirectly, RNAP2 occupancy on RDH genes. Again, for RIH genes, we did not observe such a changed pattern of RNAP2 occupancy, suggesting that this effect concerns preferentially RDH genes.

For more precise analysis, we calculated statistical significance for RPM value for PC4 OE cells relative to EBFP OE cells in three 400 nucleotide-long regions: (i) “TSS region” that covers 200 nt upstream and 200 nt downstream of TSS; (ii) “histone body” that covers 200–600 nt downstream of TSS; (iii) “3′ end” that covers 600–1000 nt downstream of TSS and is always downstream of the cleavage site (Fig. 1c). As shown in Fig. 1d, our previous observation of decreased RNAP2 occupancy in S phase and increased occupancy in G1 phase in PC4 overexpressing cells refers to all three regions of RDH genes.

**Fig. 2:**
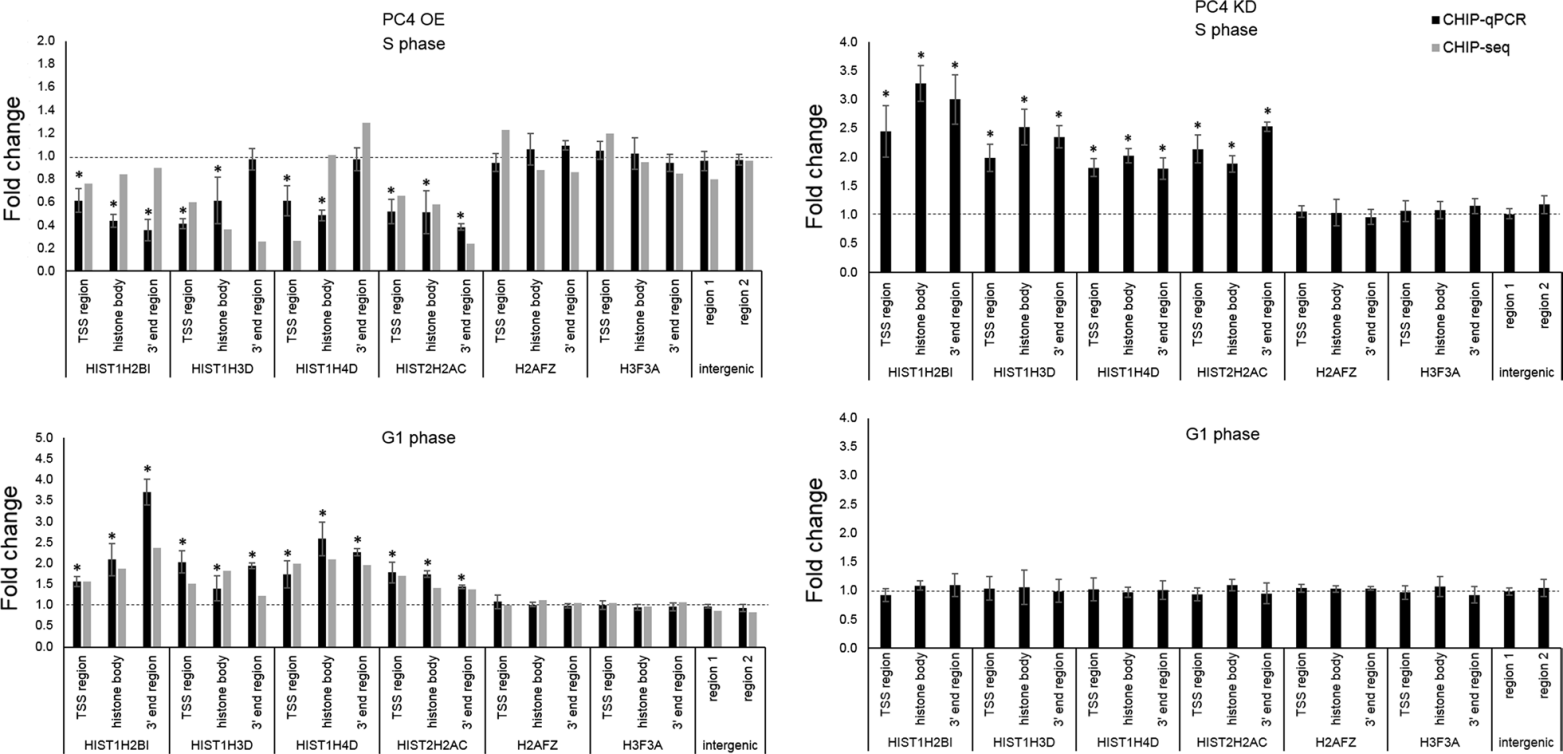
RNA polymerase II occupancy on histone genes in PC4 OE cells (left panel) and PC4 KD cells (right panel) synchronized to G1 and S phase. Analysis were done by ChIP-seq (for PC4 OE cells) and ChIP qPCR (for PC4 OE and PC4 KD cells). Charts represent mean fold change value (n = 3 for ChIP-qPCR and n = 1 for ChIP-seq). Regions are marked as described in Fig. 1c. As a negative control, two RIH genes (H2AFZ and H3F3A) as well as two intergenic regions were tested. Values were normalized to data obtained from control cells (marked by horizontal lines): EBFP OE for PC4 OE cells and HeLa scramble for PC4 KD cells. Error bars indicate standard deviations (SD) of three biological replicates. P-values were calculated on percent of input values using Student’s T-test and statistical significance is represented as follows: *P ≤ 0.05

In general, in our ChIP-seq experiments, we identified 43 and 41 genes (out of 68 RDH genes) in S phase and G1 phase, respectively, with altered RNAP2 occupancy along “TSS region”, “histone body” and “3′ end” regions in PC4 overexpressing cells. We then chose four of those genes for further investigation and confirmed ChIP-seq results by ChIP followed by qPCR as shown in Fig. 2 (left panel). As a negative control, we used two RIH genes (H2AFZ and H3F3A) and two intergenic regions. We did not observe any difference in RNAP2 occupancy in any of these control regions (Fig. 2, left panel).

Moreover, similar qPCRs were performed after ChIP experiments using HeLa cells with induced PC4 knockdown. Those cells were also synchronized to S and G1 phase (Additional file 3: Figure S2B). As expected, in case of PC4 depletion, we observed an increased RNAP2 occupancy on RDH genes during S phase. However, no significant changes were noticed in cells synchronized to G1 phase (Fig. 2, right panel). This suggests, that PC4 alters its function depending on the cell cycle and acts as transcriptional co-repressor of RDH genes transcription in S phase.

### PC4 affects RDH genes expression

To verify whether altered RNAP2 occupancy on RDH genes observed in PC4 OE and PC4 KD cells corresponds to changes in the level of replication-dependent histone mRNAs, we performed RT-qPCR using primers designed to amplify the “TSS” region, “histone body” and “3′ end” regions of previously selected RDH genes and two RIH genes, H2AFZ and H3F3A, as a negative control. All analyses were performed in PC4 OE or PC4 KD cells synchronized to S phase or G1 phase.

**Fig. 3:**
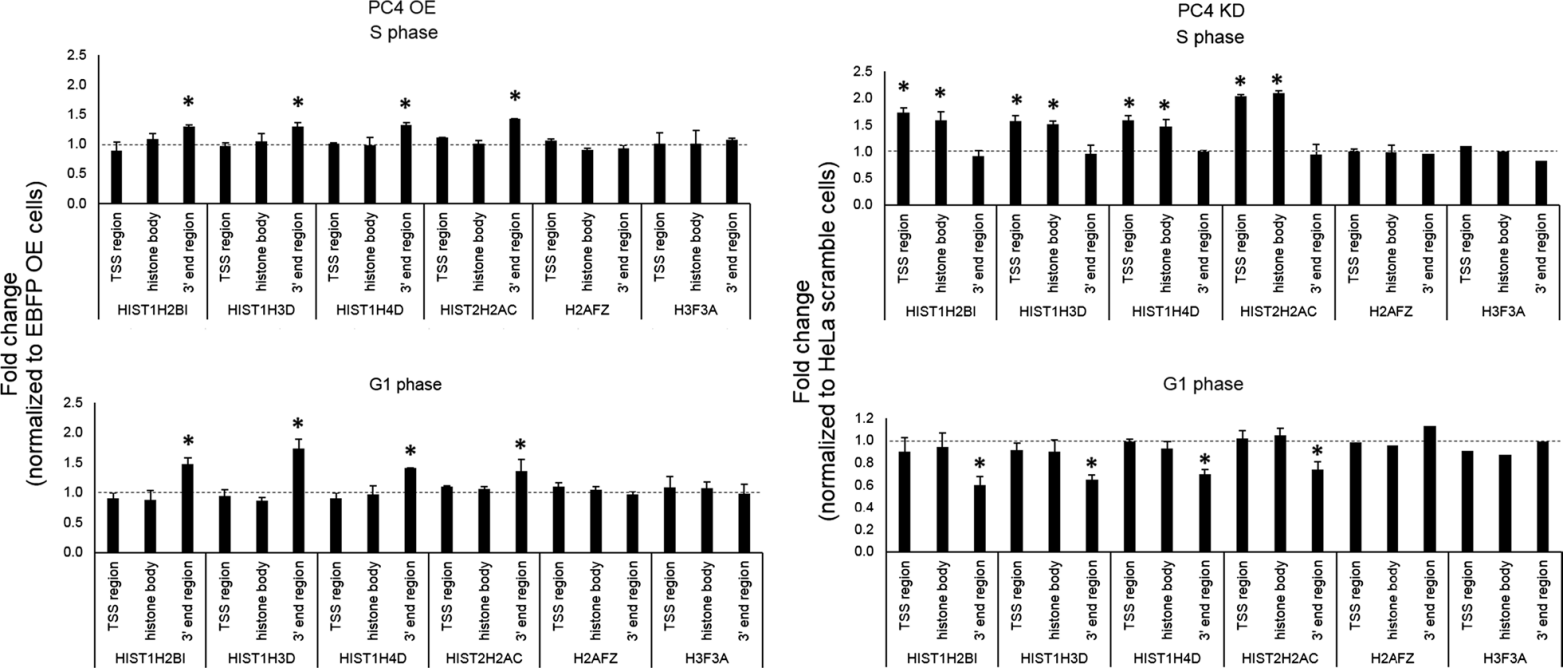
Influence of PC4 on the levels of RDH mRNAs. RT-qPCR analysis of four selected RDH (and two RIH) genes was performed in PC4 OE (left panel) and PC4 KD (right panel) cells synchronized to S or G1 phase. Regions marked as described in Fig. 1c. Error bars indicate standard deviations (SD) of three biological replicates. P-values were calculated on relative level of expression values using Student’s T-test and statistical significance is represented as follows: *P ≤ 0.05

As shown in Fig. 3 the changes in the level of RDH gene transcripts partly correspond to the changes of RNAP2 occupancy on RDH genes observed in our ChIP experiments. More specifically, we observed a significant upregulation of RDH mRNAs level (“TSS” and “histone body” regions) in cells with PC4 depletion synchronized to S phase, therefore confirming that PC4 can function as transcriptional co-repressor of RDH gene expression in this phase of the cell cycle. In contrast, but in agreement with the ChIP results, we could not detect any changes in RDH transcript levels in G1 phase-synchronized PC4 KD cells (Fig. 3, right panel).

In PC4 OE cells, we could not detect a significant effect on RDH mRNA levels corresponding to the previously observed changes in RNAP2 occupancy, be it in S or G1 phase. This could be due to the particularly high level of PC4 in PC4 OE cells (see “Discussion”). In any case, an increased RNAP2 occupancy in that condition does not translate into actual histone transcripts amounts (Fig. 3, left panel).

**Fig. 4:**
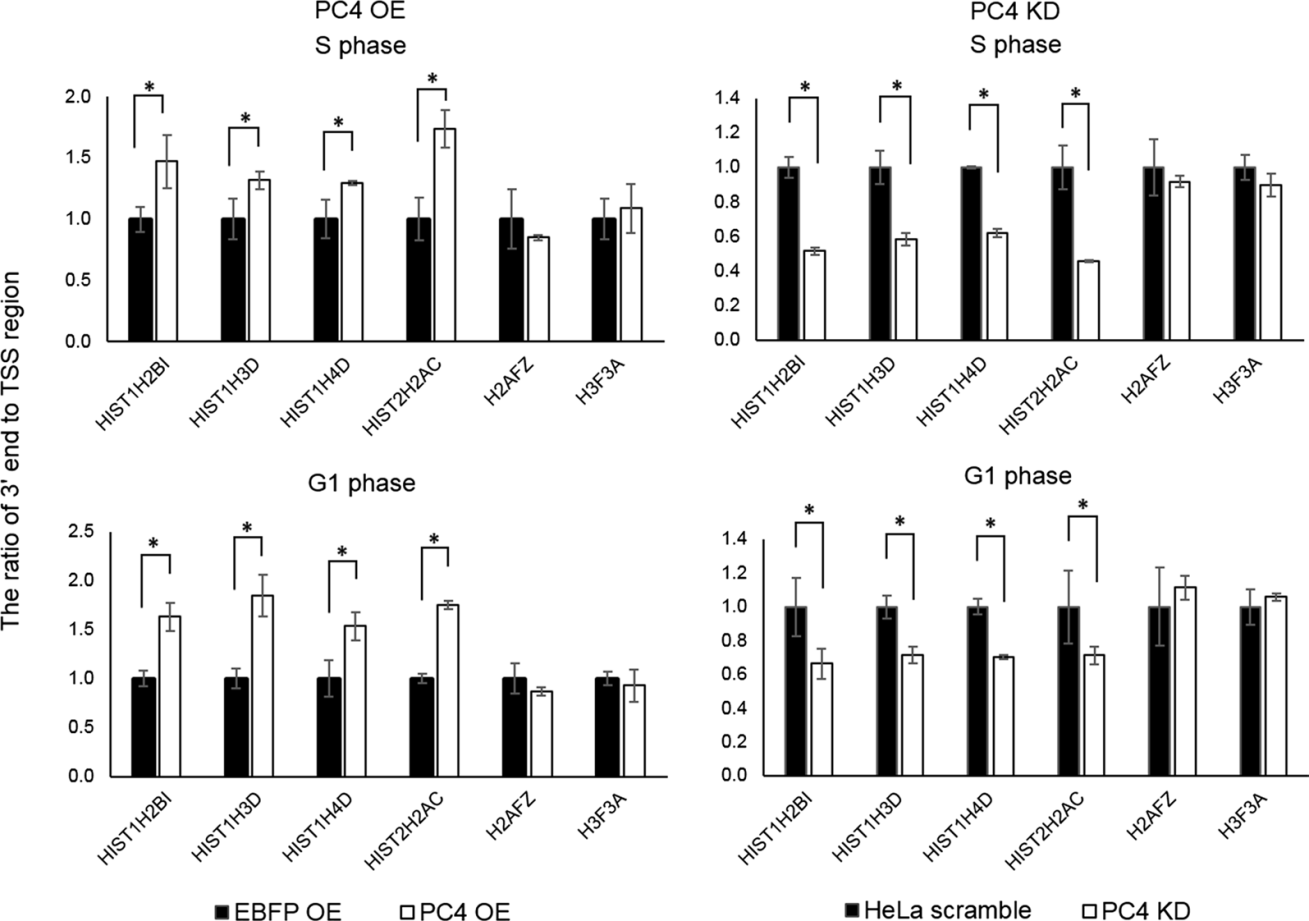
PC4 affects the apparent 3′ end processing of RDH pre-mRNAs. RT-qPCR analyses of RDH transcripts (and two RIH transcripts as controls) were performed in PC4 OE (left panel) and PC4 KD (right panel) cells synchronized to S or G1 phase. The charts represent the ratio between “3′ end” region (which is located downstream of the cleavage site and corresponds to extended transcripts) and “TSS” region (which corresponds to total mRNA level). Error bars indicate standard deviations (SD) of three biological replicates. P-values were calculated on relative level of expression values using Student’s T-test and statistical significance is represented as follows: *P ≤ 0.05

Interestingly, in both kind of cell lines (PC4 OE and PC4 KD), we observed phase-independent changes within the “3′ end” region, which is located downstream of the cleavage site and represents incorrectly processed, extended transcripts. Those transcripts results from “read-through” polymerase action and are usually polyadenylated at cryptic polyA sites [19, 28]. We then decided to test whether the apparent 3′ end processing efficiency of RDH gene transcripts might be influenced by PC4. For that purpose, we calculated the ratio of fold changes between “3′ end” and “TSS” region that correspond to the ratio between incorrectly processed transcripts and total mRNAs. As shown in Fig. 4 we noticed an elevated level of correctly processed transcripts in PC4 KD cells (right panel) and, conversely, elevated levels of extended (incorrectly processed) transcripts in cells with PC4 overexpression (left panel). The changes were observed in cells synchronized to both S and G1 phase. Again, the ratio between the levels of extended and total transcripts of RIH genes (H2AFZ and H3F3A) was not changed. These results indicate that PC4 has indeed a negative effect on the 3′ end processing of RDH genes transcripts that is independent of the cell cycle phase.

### PC4 expression and interaction during the cell cycle

**Fig. 5:**
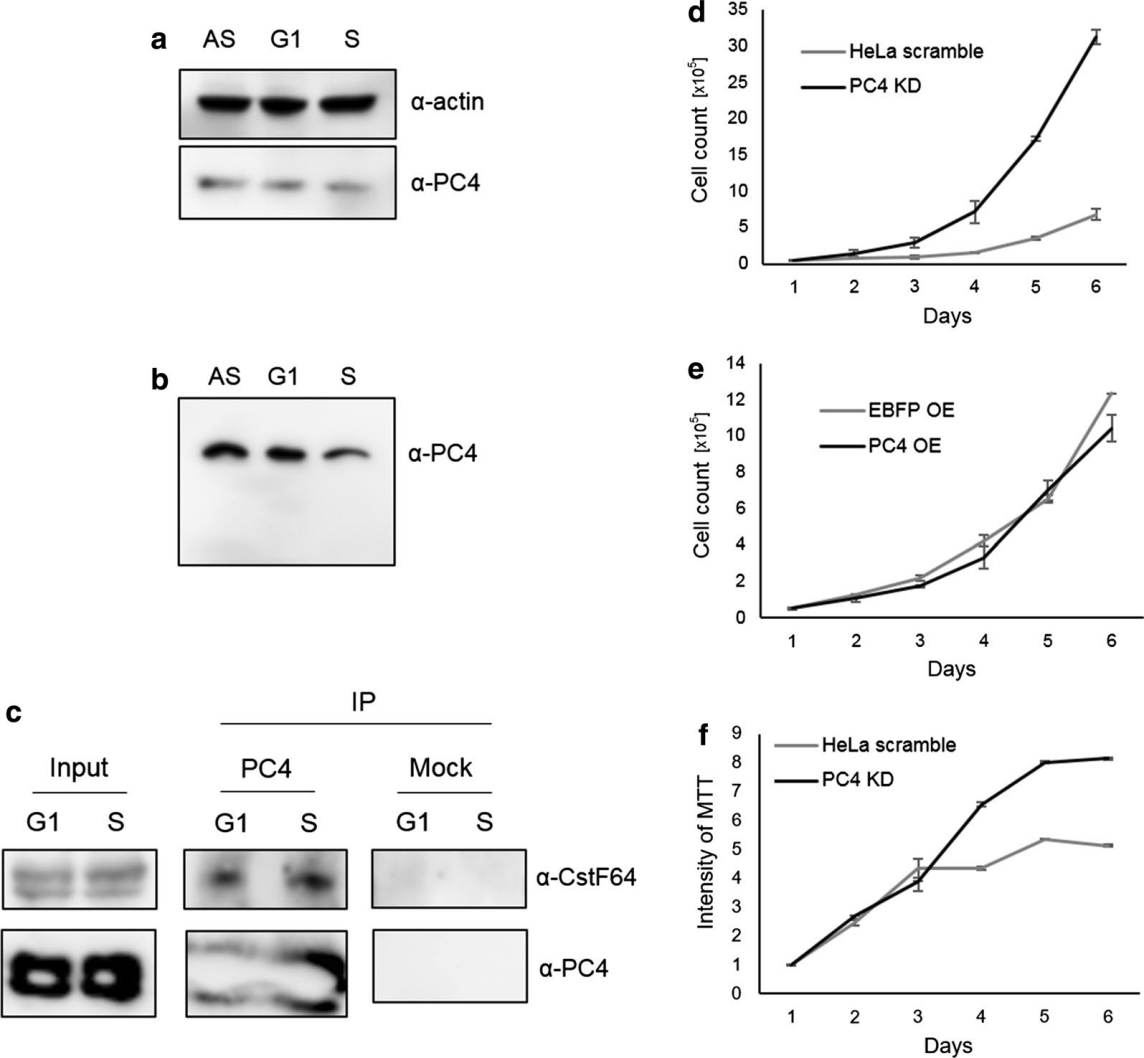
**a**–**c** PC4 protein level and its interaction with CstF64 is constant during the cell cycle. **a** Western blot followed by immunodetection with anti-actin and anti-PC4 antibodies were performed using protein extracts isolated from asynchronous HeLa cells (AS) and cells synchronized to G1 and S phase. **b** Phos-tag™-based protein electrophoresis followed by Western blot and immunodetection with anti-PC4 antibodies using protein extracts isolated from asynchronous HeLa cells (AS) and cells synchronized to G1 and S phase. **c** Protein extracts isolated from cells synchronized to G1 and S phase were subjected to immunoprecipitation with anti-PC4 antibodies conjugated to protein A-magnetic beads or non-conjugated protein A-magnetic beads (mock) followed by Western blot and immunodetection with antibodies as indicated on the right. **d**–**f** PC4 influences cell proliferation. Charts represent the mean number of cells with PC4 KD (**d**) and PC4 OE (**e**) (n = 3). Additional cell proliferation test using MTT assay was performed on PC4 KD cells (**f**). HeLa scramble and PC4 KD cells were cultured with addition of doxycycline, 1st day of experiment represents 1st day of culture with doxycycline from the day the experiment started

Considering previous results, the question arises, whether the level of PC4 protein or its posttranslational modifications (such as phosphorylation) can change during the cell cycle and thus alter its binding properties to other factors (e.g. specific transcriptional activators and/or repressors of RDH genes expression). To verify this, we first performed Western blot using protein extracts isolated from asynchronous wild type HeLa cells and cells synchronized to G1 and to S phase. As shown in Fig. 5a, the PC4 protein level is constant during the cell cycle. What is more, we could not observe any changes when we compared phosphorylation pattern of PC4 in protein extracts isolated from asynchronous or synchronized cells tested by Phos-tag™-based mobility shift detection (Fig. 5b). Moreover, PC4 interacts with CstF64 at similar strength during the cell cycle (Fig. 5c).

### PC4 depletion affects cell proliferation

As shown above, PC4 can influence the expression of RDH genes by affecting both RNAP2 occupancy on those genes and the 3′ end processing efficiency of their transcripts. Since histones are crucial for cell division, we wondered whether PC4 overexpression or depletion might also disturb cell proliferation. By seeding cells and counting them every day, we observed that PC4 depletion increases the speed of cell division (Fig. 5d). This result was confirmed in an MTT (3-(4,5-dimethylthiazolyl-2)-2,5-diphenyltetrazolium bromide) assay (Fig. 5f). However, neither accelerated cell growth nor increased histone mRNA levels observed in PC4 KD cells (Fig. 3, right panel) does change cell cycle phases distribution as one would expect (Additional file 4: Figure S3). What is more, overexpression of PC4 does not have any effect on cell proliferation (Fig. 5e). To investigate, why PC4 depleted cells divide more often, we arrested cells in G2/M transition by adding of nocodazole to the medium for 18 h. After release from the block, cells were collected every 2 h and monitored by staining with propidium iodide followed by flow cytometry analysis. However, we could not observe any significant changes in the duration of S, G1 or G2 phase in PC4 KD cells in comparison to control cells (data not shown).

## Discussion

In this paper, we describe PC4 protein as a factor that might contribute to the regulation of replication-dependent histone gene expression.

Although we did not provide direct evidence that PC4 influences RNAP2, our results based on ChIP-seq and ChIP-qPCR showed that changes in PC4 protein level resulted in altered RNAP2 occupancy on RDH genes. The most important effect was observed in S phase-synchronized cells, where PC4 knockdown leads to elevated level of RNAP2 on RDH genes, which corresponds to increased total level of RDH gene transcripts (Figs. 2, 3, right panels). The opposite effect was caused by PC4 overexpression (Figs. 2, 3, left panels). Normally, RNAP2 occupancy on RDH genes corresponds with their transcription, which is highly upregulated during S phase but stays at basal level during G2, M and G1 phases (Fig. 1a compare line EBFP OE S to EBPF OE G1). Our results suggest that in S phase PC4 may act as a co-repressor of RDH gene transcription. In light of the results obtained by us, we further hypothesize that by regulating the synthesis of histones at an optimal level, PC4 plays a role in maintaining the very delicate balance between RDH gene expression and DNA synthesis during the cell cycle. Indeed, the expression of RDH genes is inhibited when high amounts of histone synthesis is not required for DNA packaging (in G1 phase, G2 phase and replication arrest). On the other hand, even in S phase, cells need to maintain balanced synthesis of histones and DNA. Previously, it was shown that at physiological ionic strength free DNA and an excess of histones form insoluble aggregates instead of functional nucleosomes, promoting chromatin aggregation [29–31]. Therefore, PC4 might act as a negative factor that guards the cells from excess of histone proteins by repressing, directly or indirectly, RNAP2 recruitment and transcription. In accordance with the hypothesis and known data, we observed increased RNAP2 occupancy on RDH genes and significant upregulation of RDH mRNAs level in S phase, when PC4 protein level is decreased in cells. The fact that PC4 overexpression does not significantly reduce RDH mRNA level might result from extremely high level of RDH transcript in general, that compensate those PC4 OE-induced mild changes.

However, in G1 phase PC4 influence on RDH gene expression seems to be indirect. First, in cells with PC4 depletion we did not observe any effect, either in RNAP2 occupancy or in histone mRNA level. Second, in cells with PC4 overexpression histone transcript level was not altered although in this case the effect on RNAP2 occupancy on RDH genes was shown. Therefore we conclude this effect might be indirect, caused by enormously high level of PC4 in PC4 OE cells (Additional file 2: Figure S1B, C), that leads to inhibition of natural repressor of RNAP2 recruitment in G1 phase. Moreover, higher level of RNAP2 might be related to its form that is transcriptionally inactive, as in our ChIP experiments we used anti-RNAP2 antibody that recognizes both phosphorylated and unphosphorylated form of RNAP2. In that case activation or increased level of another factor that could change RNAP2 transcriptional activity would might be required.

In a very intriguing model, Calvo and Manley [20] suggest that interaction between PC4 and CstF64 links transcription, polyadenylation and termination. According to the model, at the early step of transcription, unphosphorylated PC4 interacts with transcriptional activators and general transcription factors (GTFs) that form preinitiation complex. After transcription initiation, PC4 becomes phosphorylated and dissociates from the complex. Then, during transcription elongation, the phosphorylated PC4 interacts with CstF64, which together with other factors of cleavage and polyadenylation machinery is recruited to the RNAP2. The PC4:CstF64 interaction is supposed to prevent premature transcription termination. When the termination signal is reached PC4 dissociates from CstF64, leading to transcription termination [20]. Therefore, one of our model assumed that cell-cycle regulated influence of PC4 on RDH genes expression might be due to diversified binding abilities of PC4 with CstF64 during the cell cycle. It is known that in proliferating mammalian cells the great majority of the total cellular PC4 is phosphorylated [22] and this form of PC4 interacts with CstF64 [20]—the only cell-cycle regulated factor shared between the HCC and the CPA complex [19]. In different phases of the cell cycle CstF64 can change the interacting partner, and by this way it may dictate in which type of processing complex it will be used [19]. Moreover, as phosphorylated/unphosphorylated PC4 is supposed to be associated with transcription repression/activation we hypothesized that phosphorylation status of PC4 might regulate its activity on RDH genes expression. However, in our result we could not detect any changes in PC4 protein level (Fig. 5a), phosphorylation pattern (Fig. 5b), or PC4:CstF64 interaction (Fig. 5c) during the cell cycle. Therefore, it is still not clear, how different functions of PC4 connected with RDH gene expression during the cell cycle are regulated. In one possible mechanism PC4 might act via interaction with other factors, activators or repressors, which are specific for RDH genes.

Interestingly, we found that PC4 has a negative effect on the 3′ end processing of RDH gene pre-mRNAs. We noticed an elevated level of correctly processed transcripts in PC4 KD cells and, conversely, elevated levels of extended transcripts in cells with PC4 overexpression (Fig. 4). This effect did not depend on the cell cycle. It seems like PC4 promotes the interaction of CPA complex with RDH pre-mRNAs, that in S phase might additionally impede with the recruitment of Histone Cleavage Complex. This effect is likely to be direct as polyadenylation of RIH pre-mRNAs is not significantly affected. Such a role can be played via interaction of PC4 with U7 snRNP and CstF64. In possible scenario the recruitment of HCC to the RDH pre-mRNAs mediated by CstF64 in the S phase of the cell cycle [19] is blocked by binding of PC4 to the U7 snRNP.

Finally, we found that PC4 depletion increases cell proliferation (Fig. 5d). To elucidate why PC4-depleted cells divide more often, we first analyzed the subpopulations of S-, G1-, G2-phase cells during asynchronous growing, but we could not observe any significant changes in their distribution (Additional file 4: Figure S3). Moreover, we arrested cells in G2/M by nocodazole block and then monitored their cell cycle distribution every 2 h after release from the block. However, PC4 depletion caused no obvious changes in the cell cycle transition. Therefore, we concluded that increased cell cycle proliferation in PC4 KD cells results rather from shortened cell cycle duration at all and is not related to the effect of PC4 on CstF64 and cell cycle progression. As it was previously reported, CstF64 depletion leads to inhibition of cell proliferation by their accumulation in S phase and delayed entering of G2 [19].

## Conclusions

We described novel function of positive cofactor 4 as a protein that contribute to the regulation of replication-dependent histone gene expression. We suggest that PC4 acts as a negative regulator of RNAP2 recruitment to histone gene loci in the S phase of the cell cycle in order to protect the cells from excessive transcription and synthesis of histone proteins. What is more, we observed that PC4 has a negative effect on the 3′ end processing of histone pre-mRNAs, however this effect is independent of the cell cycle phase and can be explained by the interactions of PC4 with U7 snRNP and CstF64.

Our results diversify the role of PC4, mostly known as a transcriptional co-activator. Its function in the regulation of histone gene expression is probably in cooperation with other RDH genes specific transcriptional activators and repressors. However, further analysis is necessary to describe how those interactions and function are regulated during the cell cycle.

## Methods

### Cell culture, synchronization and cell cycle analysis

HeLa cells and HEK 293T cells were grown in Dulbecco’s modified Eagle medium with l-glutamine and 4.5 g/L glucose (DMEM; Lonza) supplemented with 10 m % fetal calf serum (Gibco) and antibiotics [100 U/mL penicillin, 100 μg/mL streptomycin, 0.25 μg/mL amphotericin B (Sigma)] at 37 °C in a moist atmosphere containing 5% CO_2_.

For G1 synchronization cells were blocked first by 2 mM thymidine (Sigma-Aldrich) for 24 h, then released for 3 h, blocked again by 0.1 μM nocodazole (Sigma-Aldrich) for 12 h and collected 5–7 h after release. For S phase synchronization cells were blocked first by 2 mM thymidine for 17 h, then released for 12 h, blocked again by 400 μM mimosine (Sigma-Aldrich) for 14 h and collected 4.5–5 h after release. For detailed analysis of cell cycle progression, cells were synchronized in G2/M by addition of 200 ng/mL nocodazole (Sigma-Aldrich) to the medium for 18 h and then cells were collected and monitored every 2 h after release from the block.

For cytofluorometric analysis, cells were trypsinized, washed with phosphate-buffered saline (PBS) and fixed by dropwise addition to ice-cold 70% ethanol. On the day of staining, cells were washed twice with PBS, resuspended in propidium iodide staining solution [0.1% Triton X-100 in PBS, 0.2 mg/mL RNase A (Termo Scientific), 0.02 mg/mL propidium iodide (Sigma)] and incubated for at least 30 min at room temperature in the dark. Cell cycle profiles were analyzed by flow cytometry with a Guava easyCyte™ System (Merck Millipore) flow cytometer and the data was processed with InCyte Software (utilities from guavaSoft 3.1.1).

For cell proliferation tests, the cells were plated in triplicate in 12-well plates at the density of 50,000 cells/well. Then cell counts and viability were measured every 24 h for 6 days by using a Countess™ Automated Cell Counter (Life Technologies). For the MTT assay, cells were plated in triplicate in 24-well plates at 50,000 cells/well. To measure cell viability, Thiazolyl Blue Tetrazolium Bromide salt (Sigma M2128) in PBS was added to each well at 500 µg/mL final concentration. After 3 h of incubation, the formazan crystals were centrifuged at 300*g* for 10 min and dissolved by adding ethanol:DMSO (ratio 1:1). The absorption of the formazan solution was measured using an Infinite F200 PRO Tecan spectrophotometer at a wavelength of 570 nm. Cell viability was measured every 24 h for 6 days.

### Plasmid construction, lentiviral vector production and cells transduction

A lentiviral vector for the doxycycline-inducible PC4 knockdown was constructed by inserting annealed and kinased oligonucleotides (Additional file 5: Table S2) into the *Age*I/*Eco*RI sites of the pLKO-Tet-On plasmid (a gift from Dmitri Wiederschain; Addgene plasmid # 21915 [32]) to create pLKO-Tet-On-shPC4. The shRNA is expressed from this vector under the H1 promoter and is further converted into siRNA that targets nucleotides 170–188 of PC4 mRNA (numbering according to U12979.1, GCAGCAGAGATGATAACAT). A control lentiviral vector with an inducible shRNA scramble expression cassette was ordered from Addgene (a gift from David Sabatini; Addgene plasmid # 1864 [33]). The lentiviral expression vector encoding FLAG-tagged PC4 was constructed by amplification of the coding sequence of PC4 with a FLAG sequence added downstream of the AUG codon by using specific primers in a PCR (primer sequences available on request). FLAG-PC4 cDNA was then cloned under the EF-1 alpha promoter in into the *Mlu*I/*Sma*I sites of the pLV-tTR-KRAB-dsRed vector to create pLV-ttR-FLAG-PC4-dsRed. Lentiviral expression vector encoding enhanced blue fluorescence protein (EBFP) was prepared as described previously [27].

Virus production and HeLa transduction for cells with stable overexpression of FLAG-PC4 as well as PC4 shRNA and scramble shRNA was performed as follows: HEK 293T cells were transfected with pLKO-Tet-On-shPC4, scramble shRNA or pLV-ttR-FLAG-PC4-dsRed plasmids supplemented with packaging and envelope vector, psPAX2 and pMD2.G, respectively, by the calcium phosphate method [34]. Fresh medium was added to the cells 24 h after transfection, and lentiviral supernatants were collected 72 h after transfection. For transduction, HeLa cells were incubated with lentiviral supernatants supplemented with 4 μg/ml polybrene (hexadimethrine bromide, Sigma Aldrich) for 14 h, and then fresh medium was added. Highly RFP positive cells with FLAG-PC4 overexpression were selected by fluorescence-activated cell sorting using a BD FACS Aria™III (Becton–Dickinson) flow cytometer (cell sorter). The configuration of the flow cytometer was as follows: 100 μm nozzle and 20 psi (0.138 MPa) sheath fluid pressure. The cells were characterized by two non-fluorescent parameters: forward scatter (FSC) and side scatter (SSC), and one fluorescent parameter: yellow fluorescence (PE detector) from RFP collected using 585/42 band pass filter. For excitation, a 488 nm blue laser was employed. The flow cytometric analyses were performed using logarithmic gains and specific detectors settings (10,000 events were recorded per analysis). Data were acquired in a 4-decade logarithmic scale as area signals (FSC-A, SSC-A and PE-A) and analyzed with FACS DIVA software (Becton–Dickinson).

A sub-population P5 demonstrating high levels of yellow fluorescence (as measured by PE detector) was selected for sorting. The sort region (P5) was defined on bivariate dot plot (SSC-A vs. PE-A). Cell sorting preceded a doublets discrimination procedure which used measurements of height versus width scatter (FSC and SSC) signals, in order to discriminate single cells from conglomerates. Cells from sub-population P5 were sorted into 5 mL cytometric tubes.

HeLa cells with PC4 shRNA or scramble shRNA were selected by adding puromycin to the final concentration of 0.3 µg/mL for 7 days.

### RNA isolation, cDNA preparation, PCR and qPCR

RNA was isolated from cells by using TRIZOL reagent followed by DNAse treatment as described in [27]. First strand cDNAs were synthesized in 50 μL reactions with 4.5 μg of RNA by using 400 ng random hexamers as primers and 200 U Superscript III Reverse Transcriptase (SSIII RT, Thermo Scientific), according to the manufacturer’s protocol. PCR amplifications were carried out in 25 µL reactions containing 2.5 µL of Pfu buffer, 2 mM MgCl_2_, nucleotide mix [0.2 mM each dNTP (Roche)], 0.5 µM primers and 2 U of *Pfu* DNA Polymerase (Thermo Scientific). The samples were incubated for 30 cycles under the following conditions: 95 °C for 2 min, each cycle: 94 °C for 30 s, 55 °C for 30 s, 72 °C for 1 min. The reactions were completed by incubation for 10 min at 72 °C. For qPCR amplifications, 10 μL reaction mix contained 5 μL of Power SYBR Green PCR Master Mix (Applied Biosystems), 4 μL of 0.5 mM primers mix and 1 μL of 10× diluted cDNA template. The qPCR was performed under the following conditions: 95 °C for 10 min, followed by 40 cycles of 95 °C for 15 s, 60 °C for 1 min (QuantStudio™ 7 Flex Real-Time PCR Instrument). Primers used for qPCR are listed in Additional file 6: Table S3. The statistical significance of qPCR results was determined by Student’s T test.

### Antibodies, protein extract preparation, immunoprecipitation

The following primary antibodies were used in this work: anti-RPB2 (Abcam, ab10338), anti-β-actin (MP Biomedicals, 691001), anti-FLAG (Sigma Aldrich, A8592), anti-PC4 (Abcam, ab72132), anti-CstF64 (Santa Cruz Biotechnology, sc-28201). The following secondary antibodies were used: goat anti-rabbit IgG-HRP, goat anti-mouse IgG-HRP (Santa Cruz Biotechnology, sc-2004, sc-2005, respectively).

For total protein extract preparation, cells were harvested by trypsinization, washed with PBS, resuspended in lysis buffer (50 mM Tris–HCl pH 7.9, 150 mM NaCl, 1% NP-40, 0.5% sodium deoxycholate) and incubated for 10 min on ice. Supernatants containing total protein extracts were collected after 30 min centrifugation at 4 °C at 16,000*g*.

For affinity purification strategy protein extracts from HeLa cells expressing MS2-tagged U7 snRNA were purified on the MS2-MBP-bound resin and eluted from the resin by mild condition using 10 mM maltose. Samples were either directly submitted to mass spectrometric analysis or first separated on a SDS polyacrylamide gel and then selected bands were cut from the gel and submitted to mass spectrometric analysis, as described in [27]. In some cases, probes were first fractionated on 10–50% continuous glycerol gradients prior to affinity purification, as described in [27]. As an another approach HeLa nuclear extracts were incubated with a biotinylated 2′-*O*-methyl RNA oligonucleotide complementary to U7 snRNA followed by purification on streptavidin-coated Dynabeads, as described in [27].

For co-immunoprecipitation, an amount of 250 µg of protein extracts was immunoprecipitated for 2 h at 4 °C with 3 µg of anti-PC4 antibody previously conjugated for 1 h at 4 °C with gentle rotation with 20 µL of Dynabeads^®^ Protein A (Life Technologies) or with 20 µL of non-conjugated beads. 5% of protein extracts used for immunoprecipitation was kept in a separate tube as input. After immunoprecipitation, beads were washed three times with PBS-T and twice with lysis buffer, each time for 10 min and eluted by boiling in sample buffer (50 mM Tris–HCl pH 6.8, 10% glycerol, 2% SDS, 10 mM DTT, 0.1% bromophenol blue). After elution, the immune complexes were separated by SDS-polyacrylamide gel electrophoresis (PAGE) and transferred to polyvinylidene difluoride (PVDF) membrane (Millipore). The membrane was incubated for 2 h with primary antibodies in the presence of 2% milk and then detected by the enhanced chemiluminescence method (ECL, GE Healthcare) after incubation for 1.5 h with corresponding species-specific horseradish peroxidase (HRP)-coupled secondary antibody. For Phos-tag™-based mobility shift detection of phosphorylated PC4 protein electrophoresis was run according to manufacturer’s manual (Wako Pure Chemical Industries).

### Chromatin immunoprecipitation

A total of 12 × 10^6^ HeLa PC4 OE cells (or EBFP OE as negative control) or PC4-depleted cells (with HeLa scramble as negative control) were synchronized to G1 and S phase, as described above. Cells were trypsinized, washed with PBS and cross-linked with 1% formaldehyde for 10 min followed by quenching with 125 mM glycine for 5 min. Next, cells were washed twice with PBS and lysed in cell lysis buffer [10 mM Tris–HCl pH 8.1, 10 mM NaCl, 1.5 mM MgCl_2_, 0.5% NP-40, 1× EDTA-free protease inhibitor (Roche)] for 15 min on a rotating wheel at 4 °C and then centrifuged at 1200*g* for 5 min at 4 °C. The pellet was resuspended in nuclear lysis buffer [50 mM Tris–HCl pH 8.1, 5 mM EDTA, 0.5% sarkosyl, 1× EDTA-free protease inhibitor (Roche)] and moved to 1.5 mL DNA LoBind tubes (Eppendorf). After 10 min incubation on a rotating wheel, the nuclear lysate was sonicated with a Bioruptor^®^ Plus Sonicator (Diagenode) to generate DNA fragments between 200 and 700 bp (usually 28 cycles, at high intensity: 30 s ON/30 s OFF at 4 °C). Each time, the sizes of DNA fragments were verified by agarose gel electrophoresis. After sonication, the cell debris were removed by centrifugation at 18,000*g* for 15 min at 4 °C. From this point, samples were further processed as described [35]. Per sample, 4 µg of anti-RPB2 antibody (Abcam, ab10338) and 50 µL of a 50% slurry of Protein A-Sepharose^®^ 4B conjugate was used. One percent of the chromatin used for immunoprecipitation was kept in a separate tube as input. As modification of the protocol, a portion of the conjugate designated for chromatin pre-clearing was blocked for 1 h. Eluted samples were used for qPCR analysis; primer pairs encompassing the “TSS region”, “histone body” and “3′ end” regions of histone genes are listed in Additional file 6: Table S3. The quantitative analysis of precipitated material was shown as a fold change normalized to input and relative to HeLa EBFP OE or scramble cells. The statistical significance of qPCR results was determined by Student’s T test.

### CHIP-seq analysis

RNAP2 ChIP and input samples were used for library generation and sequencing by Illumina HiSeq 2000 system, performed by Fasteris SA (Switzerland). The quality of generated data was verified by the FastQC software [36]; libraries were mapped to the human genome (GRCh38/hg38; released 2013/12/17) by bowtie [37]; RNAP2 enriched/depleted regions were identified using MACS software; the gene annotation was done in HOMER software [38]; profiles of RNAP2 occupancy were created using bedtools package [39].

## Additional files

**Additional file 1: Table S1.** List of PC4 protein identifications by mass spectrometry.

**Additional file 2: Figure S1.** HeLa cell lines with PC4 overexpression and inducible knockdown of PC4. (A, C) RT-qPCR was performed using primers designed to amplify PC4 mRNA in control cells and cells with PC4 overexpression (PC4 OE) (A) or PC4 knockdown (PC4 KD) (C). Error bars indicate standard deviations (SD) of three biological replicates. P-values were calculated on relative level of expression values using Student’s T-test, and statistical significance is represented as follows: *P ≤ 0.05. (B, D). Western blots followed by immunodetection with anti-actin and anti-PC4 antibodies were performed using protein extract isolated from wild type HeLa cells (HeLa), PC4 OE cells (B) and PC4 KD cells with (dox+) or without (dox−) doxycycline treatment (D).

**Additional file 3: Figure S2.** Flow cytometry analysis of propidium iodide-stained HeLa cells with PC4 overexpression (A) and PC4 knockdown (B) after synchronization. Graphs represents number of cells synchronized to G1 (upper panel) or to S phase (lower panel) to Yellow-B fluorescence intensity. Grey color on the histogram symbolizes asynchronous cells.

**Additional file 4: Figure S3.** Flow cytometry analysis of propidium iodide-stained asynchronous HeLa scramble cells (A) and PC4 knockdown (B). Numbers represent mean value of cells percentage with provided standard deviation value (± SD).

**Additional file 5: Table S2.** Oligonucleotides cloned into pLKO-Tet-On plasmid used for inducible gene knockdown in HeLa cells.

**Additional file 6: Table S3.** Primers used in RT-qPCR to analyze the level of histone transcripts at “TSS region”, “histone body” and “3′ end” regions.

## Abbreviations

ChIP: chromatin immunoprecipitation
ChIP-seq: ChIP followed by high throughput sequencing
CPA: cleavage and polyadenylation machinery
FLASH: FLICE-associated huge protein
FSC: forward scatter
GTFs: general transcription factors
HCC: histone cleavage complex
HDE: histone downstream element
HLF: heat-labile processing factor
Lsm11: Sm-like protein 11
MTT: (3-(4,5-dimethylthiazolyl-2)-2, 5-diphenyltetrazolium bromide)
NPAT: nuclear protein, ataxia-telangiectasia locus
PC4: positive cofactor 4
RDH: replication-dependent histones
RIH: replication-independent histones
RNAP2: RNA polymerase II
RPM: reads per million
SSC: side scatter
TSS: transcription start sites
U7 snRNP: U7 small nuclear ribonucleoprotein
