# Supplementary material for: Positive cofactor 4 (PC4) contributes to the regulation of replication-dependent canonical histone gene expression
Source: BMC Mol Biol. 2018 Jul 27;19:9. doi: 10.1186/s12867-018-0110-y (PMC6062981; doi:10.1186/s12867-018-0110-y)
Supplement: Supplementary file 1 — Additional file 1: Table S1. List of PC4 protein identifications by mass spectrometry. [file 12867_2018_110_MOESM1_ESM.pdf]

***Additional file 1: Table S1***. *List of PC4 protein identifications by mass spectrometry.*

| PMSS* | Seq. cov. (%) | Sample analyzed |
|---|---|---|
| 65.8 | 26 | 1xMS2-purified |
| 48.6 | 26 | 3xMS2-purified |
| 13.0 | 19 | 3xMS2-purified |
| 16.4 | 19 | negative control sample |
| 72.2 | 32 | gradient, then biotin-purified |
| 39.4 | 26 | gradient, then biotin-purified |
| 22.7 | 19 | gradient, then biotin-purified |
| 37.4 | 31 | gradient, then biotin-purified |

*PMSS score is a measure for semiquantitative protein abundance; 1xMS2(3xMS2)-purified sample: fraction eluted after affinity purification of 1xMS2(3x-MS2)-labeled U7 snRNA; biotin-purified: fraction eluted after pull down using antisense biotinylated oligonucleotide; gradient: protein extract was fractionated on 10-50% glycerol gradient and selected fractions were affinity-purified as indicated.
