# Supplementary material for: Positive cofactor 4 (PC4) contributes to the regulation of replication-dependent canonical histone gene expression
Source: BMC Mol Biol. 2018 Jul 27;19:9. doi: 10.1186/s12867-018-0110-y (PMC6062981; doi:10.1186/s12867-018-0110-y)
Supplement: Supplementary file 2 — Additional file 2: Figure S1. HeLa cell lines with PC4 overexpression and inducible knockdown of PC4. (A, C) RT-qPCR was performed using primers designed to amplify PC4 mRNA in control cells and cells with PC4 overexpression (PC4 OE) (A) or PC4 knockdown (PC4 KD) (C). Error bars indicate standard deviations (SD) of three biological replicates. P-values were calculated on relative level of expression values using Student’s T-test, and statistical significance is represented as follows: *P ≤ 0.05. (B, D). Western blots followed by immunodetection with anti-actin and anti-PC4 antibodies were performed using protein extract isolated from wild type HeLa cells (HeLa), PC4 OE cells (B) and PC4 KD cells with (dox+) or without (dox−) doxycycline treatment (D). [file 12867_2018_110_MOESM2_ESM.pdf]

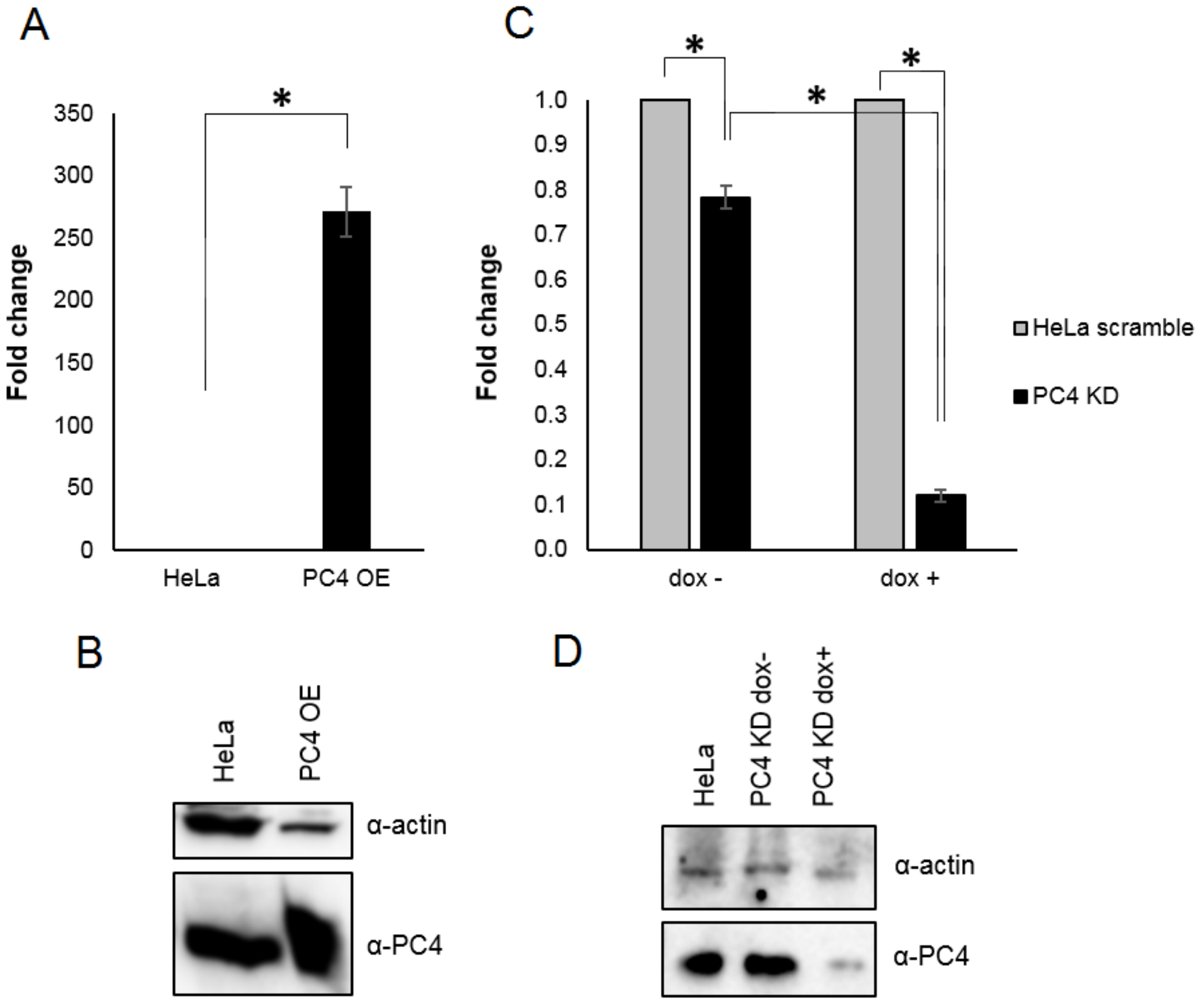

Additional file 2: Figure S1*. HeLa cell lines with PC4 overexpression and inducible knockdown of PC4. (A, C) RT-qPCR was performed using primers designed to amplify PC4 mRNA in control cells and cells with PC4 overexpression (PC4 OE) (A) or PC4 knockdown (PC4 KD) (C). Error bars indicate standard deviations (SD) of three biological replicates. P-values were calculated on relative level of expression values using Student's T-test, and statistical significance is represented as follows: *p $\leq$ 0,05. (B, D). Western blots followed by immunodetection with anti-actin and anti-PC4 antibodies were performed using protein extract isolated from wild type HeLa cells (HeLa), PC4 OE cells (B) and PC4 KD cells with (dox+) or without (dox-) doxycycline treatment (D).*
