# Supplementary material for: Positive cofactor 4 (PC4) contributes to the regulation of replication-dependent canonical histone gene expression
Source: BMC Mol Biol. 2018 Jul 27;19:9. doi: 10.1186/s12867-018-0110-y (PMC6062981; doi:10.1186/s12867-018-0110-y)
Supplement: Supplementary file 3 — Additional file 3: Figure S2. Flow cytometry analysis of propidium iodide-stained HeLa cells with PC4 overexpression (A) and PC4 knockdown (B) after synchronization. Graphs represents number of cells synchronized to G1 (upper panel) or to S phase (lower panel) to Yellow-B fluorescence intensity. Grey color on the histogram symbolizes asynchronous cells. [file 12867_2018_110_MOESM3_ESM.pdf]

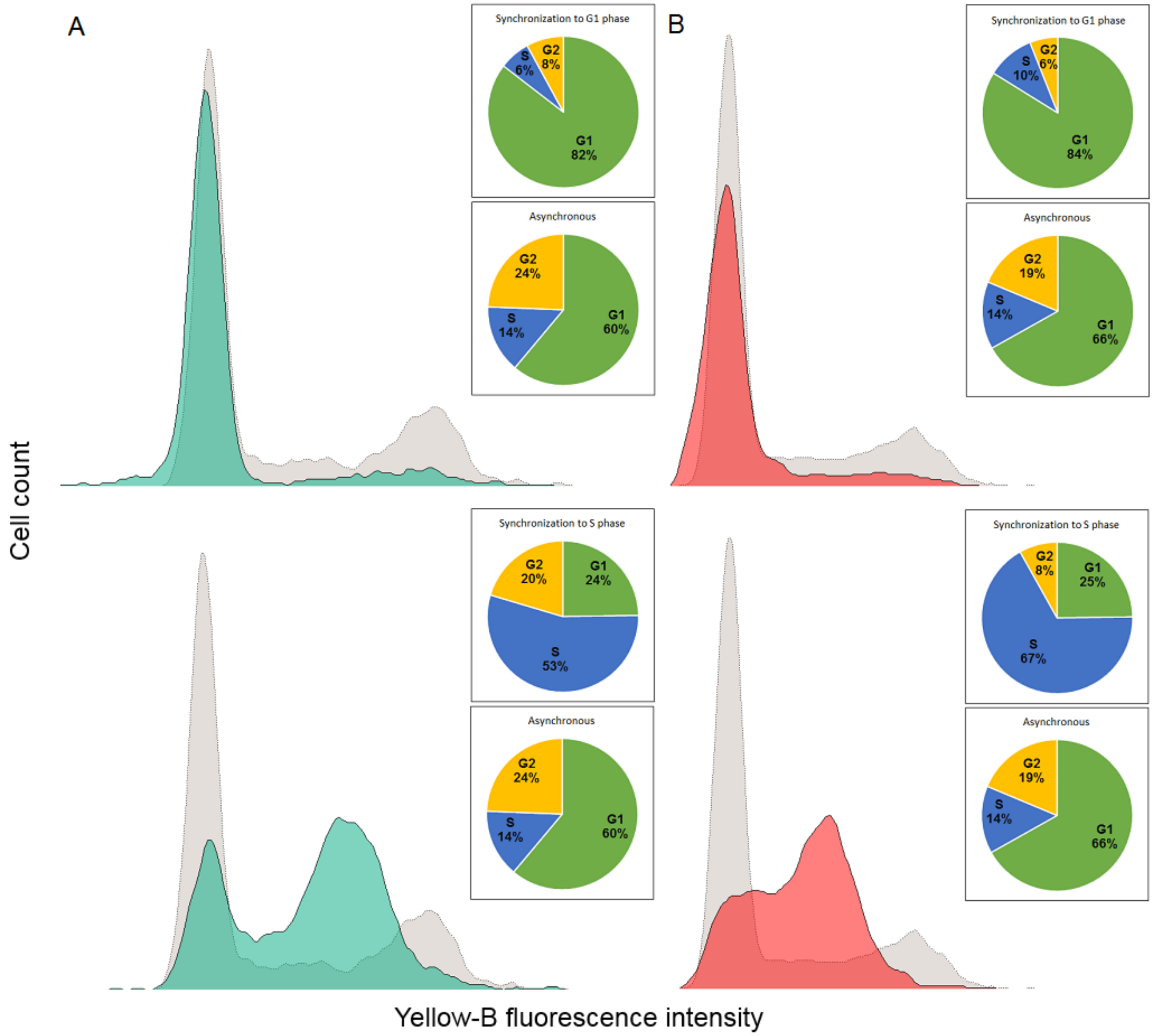

Additional file 3: Figure S2. *Flow cytometry analysis of propidium iodide-stained HeLa cells with PC4 overexpression (A) and PC4 knockdown (B) after synchronization. Graphs represents number of cells synchronized to G1 (upper panel) or to S phase (lower panel) to Yellow-B fluorescence intensity. Grey color on the histogram symbolizes asynchronous cells.*
