# Supplementary material for: Positive cofactor 4 (PC4) contributes to the regulation of replication-dependent canonical histone gene expression
Source: BMC Mol Biol. 2018 Jul 27;19:9. doi: 10.1186/s12867-018-0110-y (PMC6062981; doi:10.1186/s12867-018-0110-y)
Supplement: Supplementary file 4 — Additional file 4: Figure S3. Flow cytometry analysis of propidium iodide-stained asynchronous HeLa scramble cells (A) and PC4 knockdown (B). Numbers represent mean value of cells percentage with provided standard deviation value (± SD). [file 12867_2018_110_MOESM4_ESM.pdf]

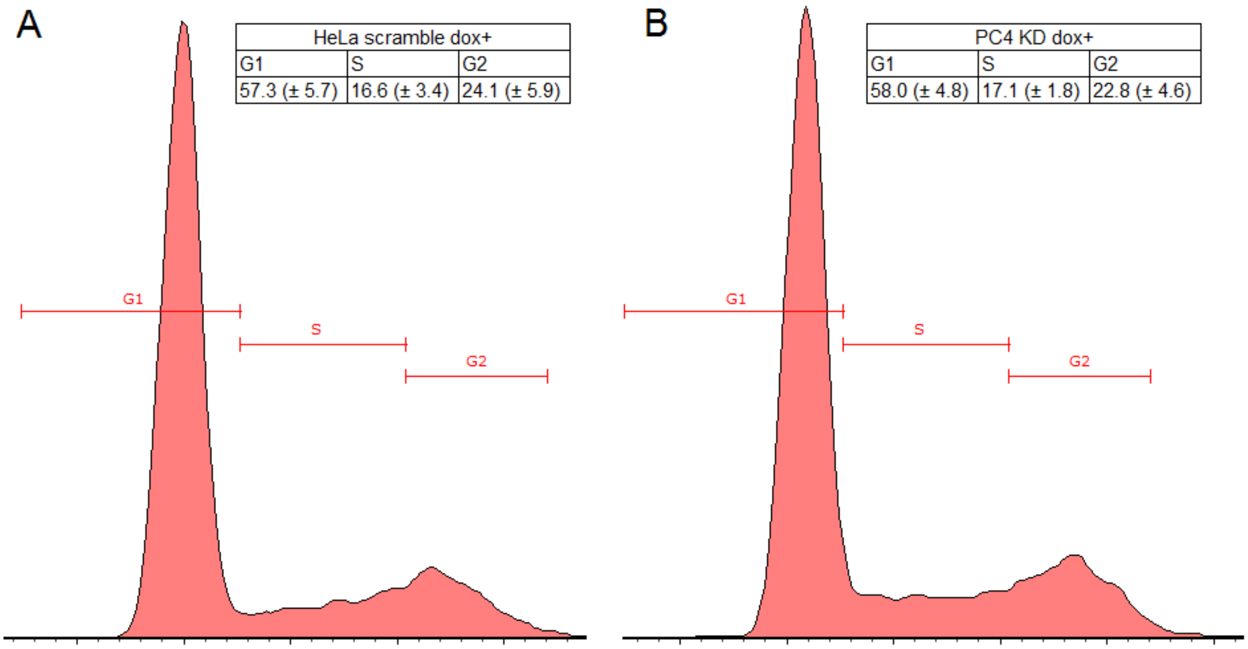

Additional file 4: Figure S3*. Flow cytometry analysis of propidium iodide-stained asynchronous HeLa scramble cells (A) and PC4 knockdown (B). Numbers represent mean value of cells percentage with provided standard deviation value (± SD).*
