# Supplementary material for: Positive cofactor 4 (PC4) contributes to the regulation of replication-dependent canonical histone gene expression
Source: BMC Mol Biol. 2018 Jul 27;19:9. doi: 10.1186/s12867-018-0110-y (PMC6062981; doi:10.1186/s12867-018-0110-y)
Supplement: Supplementary file 5 — Additional file 5: Table S2. Oligonucleotides cloned into pLKO-Tet-On plasmid used for inducible gene knockdown in HeLa cells. [file 12867_2018_110_MOESM5_ESM.pdf]

Additional file 5: Table S2*. Oligonucleotides cloned into pLKO-Tet-On plasmid used for inducible gene knockdown in HeLa cells.*

**Target mRNA**	**Description**	**Sequence 5'-3'**
scramble	*sense strand*	*CCGGT**CCTAAGGTTAAGTCGCCCTCG**CTCGAGCGAGGGCGACTTAACCTTAGGTTTTT*
	antisense strand	*CCTAAGGTTAAGTCGCCCTCGCTCGAGCGAGGGCGACTTAACCTTAGG*
PC4	*sense strand*	*CCGG**GCAGCAGAGATGATAACAT**CTCGAGATGTTATCATCTCTGCTGCTTTTT*
	antisense strand	*AATTAAAAAGCAGCAGAGATGATAACATCTCGAGATGTTATCATCTCTGCTGC*

The sequences of siRNA are marked.
