# Supplementary material for: Positive cofactor 4 (PC4) contributes to the regulation of replication-dependent canonical histone gene expression
Source: BMC Mol Biol. 2018 Jul 27;19:9. doi: 10.1186/s12867-018-0110-y (PMC6062981; doi:10.1186/s12867-018-0110-y)
Supplement: Supplementary file 6 — Additional file 6: Table S3. Primers used in RT-qPCR to analyze the level of histone transcripts at “TSS region”, “histone body” and “3′ end” regions. [file 12867_2018_110_MOESM6_ESM.pdf]

Additional file 6: Table S3*. Primers used in qPCR to analyze the level of histone transcripts at "TSS region", "histone body" and "3' end" region.*

Gene name	Amplified region	Primer sequence 5'-3'
H3F3A	TSS	F: GCCATCTTTCAATTGTGTTCGC
		R: CCTTCTCCTTCGGCTGG
	histone body	F: CAAGTGAGGCCTATCTGGT
		R: TAAGCACGTTCTCCACGT
	3' end	F: CTAATTTTTAGAAGCTTGCCACT
		R: AAGTTCAATCTACTTACAGATTCCA
H2AFZ	TSS	F: GCTGGCGGTAAGGCTG
		R: CTGCAAGCCGGCTCTC
	histone body	F: GTGTCATTCCACACATCCAC
		R: CTGGACAGCTGTTAGAGTATTTAG
	3' end	F: CTAGCAGCAATAACTAAGCAC
		R: GCCTAAGAAACTGACAATCC
HIST1H2BI	TSS	F:CTGAACCAGCTAAGTCAGCTCCC
		R: GTTCATAATCCCCATAGCCTTGGAC
	histone body	F: GAGATCCAAACGGCTGTGCG
		R:GTCGTTAGCGCTTTTACCCAGG
	3' end	F: TGTAAGCGTCACCAAGGAGT
		R: TTAAGATGGTGGTGTGCGGG
HIST1H3D	TSS	F: CGGGTGGGAAAGCGCCA
		R: CGTGCCGGGCCGGTAA
	histone body	F: AGGACTTCAAGACTGATCTGCGTTTT
		R: CTTGGCGTGAATGGCGCAT
	3' end	F: GCCACCTTTGACCAACCAAC
		R: AGCTTGGGGCGTCCTTATTC
HIST1H4D	TSS	F: GCGGAAAGGGTCTAGGTAAGGGT
		R: CTTTCAGCACTCCGCGAGTT
	histone body	F: CGATGCTGTCACCTACACGGAA
		R: TGGCGCTTGAGCGCGTAC
	3' end	F: GCTTCATATCTTACTGGCCGGTGA
		R: GGAGACTGCAGATTGCCGAGATC
HIST2H2AC	TSS	F: CCTCCAGTTCCCGGTAGGG
		R: CCGCCGCCATGTAGACG
	histone body	F: GCGCATCATCCCTCGTCACCTC
		R: GTGGCTTTCGGTTTTCTTTGGTAACAGA
	3' end	F: GAGCGCGACTTGGCCTTA
		R: GCAATTTTAGCCGTAATGTCAGGA
intergenic region 1		F: TGCTGGGATTACAGGTGTGAGC
		R: ATTCTGGGGACATCTTCAGGAACTCA
intergenic region 2		F: TGCTGATAATACTGCTACGAAGGCTG
		R: TTTGTGGTTCATCTTTTGAAGTTTCTTTGAGT
PC4	RT-qPCR	F: GGCAGTGATTCTGACAGTGAGGTTG
		R: CTGCTGCTGCTGCTCTGTTTAG
F, forward primer, R reverse primer		
